# Life Course Pathways From Childhood SES to Age-Related Declines in Kidney Function Across Adulthood

**DOI:** 10.1093/geroni/igaa057.3044

**Published:** 2020-12-16

**Authors:** Agus Surachman, Alexis Santos, Jonathan Daw, Lacy Alexander, Christopher Coe, David Almeida

**Affiliations:** 1 The Pennsylvania State University, University Park, Pennsylvania, United States; 2 Pennsylvania State University, University Park, Pennsylvania, United States; 3 University of Wisconsin - Madison, Madison, Wisconsin, United States

## Abstract

Age is a strong predictor of declines in kidney function across adulthood. Using data from 2,045 adults (ages 25-84) in the Midlife in the United States (MIDUS) study, we examined the life course pathways through which low parental education, through adult SES and body mass index (BMI), was associated with faster age-related declines in kidney function. Kidney function declines by 0.8 mL/min/1.73 m2 per year across adulthood. Lower parental education, through adult SES and BMI, was associated with higher kidney function among younger adults (Est = -1.61, SE = 0.62, 95%CI = -2.62, -0.60), but lower kidney function among older adults (Est = 0.93, SE = 0.51, 95%CI = 0.11, 1.79). The impact of early socioeconomic adversity on kidney function is initiated by kidney hyperfiltration in early adulthood and followed by faster declines and development into disease state in later adulthood.

